# Managing Fear Responses: A Qualitative Analysis of Pictorial Warning Labels Five Years Post-Plain Packaging

**DOI:** 10.1093/ntr/ntae112

**Published:** 2024-06-06

**Authors:** Ellen Ozarka, Lani Teddy, Mei-Ling Blank, Andrew Waa, Janet Hoek

**Affiliations:** Department of Public Health, University of Otago, Wellington, Aotearoa, New Zealand; Te Rōpū Rangahau Hauora a Eru Pōmare Department of Public Health, University of Otago, Wellington, Aotearoa, New Zealand; Department of Preventive and Social Medicine, University of Otago, Dunedin, Aotearoa, New Zealand; Te Rōpū Rangahau Hauora a Eru Pōmare Department of Public Health, University of Otago, Wellington, Aotearoa, New Zealand; Department of Public Health, University of Otago, Wellington, Aotearoa, New Zealand

## Abstract

**Introduction:**

Although pictorial warning labels (PWLs) now dominate tobacco packages sold in many countries, few studies have probed how people who smoke respond to the threats presented several years post-plain packaging and larger PWLs. Understanding how people manage the fear and dissonance PWLs arouse, and the strategies they use to rationalize, diminish, and reject risk messages, could inform future PWL design.

**Aims and Methods:**

We undertook 27 in-depth interviews with people aged 18 and over (16 female, 8 Māori, and 13 aged ≤35) who smoked roll-your-own tobacco and lived in Aotearoa New Zealand. We probed participants’ views on current PWLs and how they responded to these, then asked them to use alternative images and headlines to create new PWLs. We drew on the extended parallel processing model to interpret the data, which we analyzed using a reflexive thematic analysis approach.

**Results:**

People who smoke dislike PWLs, which they think reduce them to diseased body parts. While a minority thought existing PWLs were believable and effective, most reported avoiding PWLs by hiding or cognitively blocking them. Participants used diverse counterarguments to diminish PWLs’ relevance and impact, and a minority displayed strong reactance. Several suggested developing PWLs that recognized them as whole people rather than patients in waiting, and recommended greater use of testimonials, particularly from people who had successfully become smoke free.

**Conclusions:**

PWLs using more holistic and diverse messages could elicit greater engagement and responsiveness, and motivate cessation more effectively than existing health-oriented warnings.

**Implications:**

Our findings suggest existing PWLs, which aim to arouse fear of ill health, could be complemented by warnings that emphasize the benefits of quitting. Continuing to use threat-based PWLs could stimulate greater rationalization and reactance. By contrast, PWLs that aim to illustrate how cessation could benefit people who smoke and their families, rather than instill a fear of disease, could avoid message rejection and counter-argument, and may prove a more powerful way of motivating cessation.

## Introduction

Article 11 of the Framework Convention on Tobacco Control recommends signatories require all smoked tobacco products to display pictorial warning labels (PWLs).^1^ In 2001, Canada became the first country to introduce PWLs^2^; more than 20 nations have now gone further and implemented “plain packaging,” a policy that replaces tobacco branding with a dissuasive green color and larger PWLs.^3^

PWLs aim to increase awareness and understanding of smoking’s health risks,^4^ and typically feature striking visual imagery that arouses strong negative emotions, such as fear.^5^ Evaluations show PWLs capture the attention of people who smoke, motivate cessation-related behaviors such as foregoing and delaying cigarette consumption, and prompt quit attempts more effectively than text only warnings.^5–8^ Plain packaging provided an opportunity to increase PWLs’ size, which several studies have concluded increased PWLs’ impact.^9–11^ Studies from Canada and Australia found this policy had reduced smoking prevalence beyond business as usual measures.^12,13^

Researchers have theorized that people respond to PWLs according to how they perceive the *severity* of the risk shown and their own personal *susceptibility* to that risk.^14–16^ Responses also depend on how people assess their ability to manage the threat presented (ie, their self-efficacy to quit smoking or seek health screening) and the likely outcome (ie, response efficacy).^15^

The extended parallel processing model (EPPM)^16^ combines earlier models outlining these pathways and explains how adaptive or maladaptive responses to fear-arousing stimuli, such as PWLs, arise. Where people perceive a serious but manageable threat, they experience an adaptive danger control response and act to reduce the threat. However, if a threat appears too strong, or people view their coping ability as too low, they will experience a maladaptive fear control response.^16^ For example, they may deny or minimize the risk presented by assessing the PWL as inaccurate or irrelevant, reviewing it more critically, or giving greater weight to alternative explanations.^17^ They may also try to avoid the threat (eg, minimize their exposure to PWLs), become defensive (eg, provide alternative explanations), or display reactance (eg, reaffirming the behavior challenged by derogating the PWL).^17–20^

### Minimizing exposure

Several studies have examined how people minimize exposure to PWLs, such as by requesting alternative packs (ie, with less disturbing images) from retailers.^10^ Eye-tracking studies conducted prior to plain packaging found that people refocused their attention, with those who smoke daily more likely than those smoking less frequently to avoid looking at PWLs.^21^ Post-implementation of plain packaging, people who smoked daily gave greater attention to PWLs than to branding.^22^ While this latter finding accords with observational studies,^23^ attention may decline as familiarity with plain packaging increases and wear out occurs.^24^

Other avoidance strategies include decanting cigarettes or tobacco into alternative containers, or using pack sleeves to obscure the PWL.^25^ Studies undertaken post-plain packaging found increased reports of pack covering as people who smoke became more likely to think they should cover their packs and less likely to leave these on display.^10,26–28^

While avoiding exposure seems intuitively likely to reduce PWLs’ impact, these strategies are actually associated with increased cessation-related behaviors, including more frequent thoughts about smoking’s harms and forgoing cigarettes.^29^ Deliberate efforts to avoid exposure to PWLs may thus indicate greater engagement with the message content and increased impact.^19,30^

### Defensiveness and Counterargument

PWLs have also elicited defensiveness, where people attribute the risks presented to factors other than smoking, or argue the information featured is inaccurate, irrelevant, or less compelling than alternative explanations.^17^ Other strategies to discount personal susceptibility include challenging warning truthfulness and accuracy, or self-exempting from the harms shown.^25,31,32^ However, very few studies have probed defensive reactions post-plain packaging or explored how counterarguments evolve.^25,26^

### Reactance: Asserting Freedoms

Reactance occurs when people reject perceived limits on their “freedoms”^6,33–35^; for example, if they interpret PWLs as coercive. Studies exploring reactance to PWLs found these were more likely than text-only warnings to elicit reactance^20,35,36^ or affective responses, such as anger,^33,37^ though we found no studies exploring reactance post-plain packaging.

Many studies have examined responses to PWLs, though fewer have done so several years post-implementation, when wear out may have diminished their effectiveness. Most examine avoidance rather than other maladaptive responses,^23,38^ and very few qualitative studies have systematically probed fear management strategies.^25,39^ Furthermore, earlier studies have focused almost exclusively on responses to tailor-made cigarette packaging and paid less attention to roll-your-own (RYO) pouches (with some exceptions^40,41^), despite evidence that RYO users perceive this tobacco as less harmful, and may thus exhibit different responses to PWLs.^42^ Aotearoa/NZ provides a unique context for exploring these questions as RYO use is high relative to other countries and PWLs have not been updated since their introduction in 2018. We thus addressed the following research questions:

1. How do people smoking RYO tobacco interpret and respond to PWLs five years post-plain packaging?
2. What strategies do they use to manage fear aroused by PWLs?
3. How could PWLs become more effective?

## Methods

### Participants

Participants were eligible if they were aged at least 16, lived in Ōtepoti Dunedin or Te-Whanganui-a-Tara Wellington (2 large cities within Aotearoa, New Zealand) and could attend an in-person interview on the University campus. Smoking-related eligibility criteria included having smoked at least 100 cigarettes and currently smoking at least once a week, with RYO tobacco accounting for at least half the cigarettes smoked.

JH, EO, and LT recruited 27 participants using social and community media, and personal networks. We used convenience sampling approaches but managed recruitment purposively to maximize Māori participants and achieve a reasonable gender balance. Interested people could obtain information via phone or email, or proceed to an online eligibility survey (Supplementary File 1). We provide a recruitment flowchart in Supplementary File 2. All participants gave verbal consent and received a $40 voucher to recognize their time and goodwill. The University of Otago Human Ethics Committee reviewed and approved the project (reference 19/122).

### Interviews

JH, EO, and LT conducted individual in-person interviews between January 2023 and May 2023. We began with whakawhānaungatanga, a relationship-building process; where participants wished, we said a karakia (secular prayer used to open and close meetings) and then introduced themselves, outlined details of our background and invited participants to respond. This process helps identify points of connection and creates a safe space for participants to share personal information (Supplementary File 3 contains the interview guide).

Using a semi-structured interview guide, we probed participants’ experiences of smoking, adoption of RYO tobacco, and views on current PWLs. In an elicitation exercise, we probed responses to potential new PWL components and efficacy messages, which participants used to design a new RYO pouch.^40^ In this MS, we report on responses to existing and potential PWLs.

Interviews lasted between 44 and 93 minutes. With participants’ permission, we audio-recorded all interviews and used an online transcribing service to provide verbatim records, which we checked and corrected manually. We assigned each participant a pseudonym and sent them a copy of their transcript and summary notes, which they could edit (none did).

### Data Analysis

Although we used the EPPM as a guiding theory, we interpreted the data using an inductive reflexive thematic analysis approach that recognized our status as health researchers with varying training and world views. Our constructivist approach explored participants’ responses and sense-making processes^43,44^; to foster reflection, we wrote analytical memos to document our evolving interpretations.

EO, JH, and LT independently read three transcripts and identified preliminary codes. As a Māori researcher, LT reviewed transcripts from Māori participants and considered whether these contained new themes or sub-themes specific to Māori. She found no consistent differences across ethnicities that required nuanced analyses. We discussed our ideas in a workshop, prepared a detailed framework, and began developing themes. EO used this framework to code the remaining transcripts; we met frequently during this process to review new themes and sub-themes. JH reviewed the entire codebook for consistency and she, EO and LT discussed and resolved queries that arose during that process. We used NVivo 20 (Release 1.7.1) to code the data and set out high-level themes using Miro, a digital whiteboard tool.

## Results

We interviewed 27 people in Dunedin and one in Wellington. We summarize participants’ demographics and smoking behaviors in Table 1, then present and disambiguate the themes identified. We identify each participant by pseudonym, age and ethnicity.

**Table 1.** Sample Demographics and Smoking History

Attribute	Total
Gender	
Female	16
Male	11
Ethnicity	
Māori	8
NZ European	18
Asian	1
Age	
< 35	13
35+	13
No data^1^	1
Years smoking	
< 10	5
10–19	8
20+	12
No data	2
Smoking frequency	
Daily	26
Weekly	1
Current smoked tobacco product preference	
Only roll your own	24
Both roll your own and tailor made cigarettes	3
Age when regular smoking commenced	
<20	23
20+	4
Cigarettes per day^2^	
<10	7
10–19	9
20+	11
Heaviness of smoking index score^45^	
Low addiction	10
Moderate addiction	13
High addiction	4
1. One participant declined to answer some demographic questions. 2. Either RYO or tailor made cigarettes	

Participants’ views on PWLs varied and often conflicted. A minority thought PWLs “*really hit you*” (Amber/62/NZE), were “*believable*” (Maia/29/M) and “*would have to be factual to be allowed to go through*” (Rawiri/68/M). Some recognized symptoms they had experienced; Maia was *“already starting to see signs of that* [aging]*... I’m only 29”* and explained how her brother “*…got asthma from* [their mother’s] *smoking, and I’ve seen him in hospital”* (29/M). A PWL on blindness resonated with Tui *“because… I have to have glasses or contacts in to see, which I feel is also brought on from smoking,” and she reflected on a baby image noting: “I had a miscarriage... that’s why this one stands out … the most”* (28/M). However, despite these connections, most participants managed the negative affect or dissonance PWLs aroused using avoidance and counterarguments, and some displayed reactance. For example, some turned over images of existing PWLs or relocated these away from their line of sight. Although many identified flaws in existing PWLs, they nonetheless felt these could be effective and suggested changes they felt would elicit adaptive responses, such as increasing quit intentions.

We drew on the EPPM to probe maladaptive and fear management strategies participants used to diminish the negative affect PWLs elicited and the resulting pressure to change their beliefs or quit intentions. We present the themes below and provide additional quotations in Supplementary File 4.

### Avoidance

As the EPPM implies, avoidance reduces the negative affect PWLs arouse and several participants reported using avoidance strategies. Amber would “*just turn my packet over and think ‘well, that’s not happening, so I’m all right*’” (62/NZE); out-of-sight was not simply out-of-mind but an opportunity to reassert her invulnerability to smoking-related harms. Maia knew “*people that have taken the plastic cover off and got a vivid and scribbled it out because they haven’t wanted to see it*” (29/M). Changes in pack design, such as zip locks, meant participants could easily remove and avoid PWLs, as Ryan conceded: “*All that gets ripped off. ‘Cause of the extra packaging… And then it’s just no flap and it’s only that* [i.e. pouch with no PWL]*. But not for the reason, not to get rid of the pic-... well, yeah, in a way to get rid of the pictures, ‘cause they’re disgusting*” (51/NZE). Participants also used containers to hide PWLs. Cara “*…made a pouch to put them into so I didn’t have to look at them*” (55/NZE) while Victor decanted his tobacco into a tin: “*before, when you seen me open* [the tin] *up, it’s not in your face”* (56/NZE).

Familiarity facilitated avoidance, which had become easier over time. Victor noted: “*when the pictures first come out on the cigarettes… for a while it was quite effective. People would go, ‘Oh’…”* (56/NZE). However, he went on: “*I think it’s just sort of worn off as such where people aren’t so taken by the pictures*.” Cara agreed and felt people had become “*hardened”* to PWLs: “*Over time… they’ve become a part of the packaging of our cigarettes. So, we just, kind of, switched off a bit…. When you see it a thousand times… it doesn’t have any meaning”* (55/NZE).

Perceptions of PWLs’ diminishing impact helped participants disengage and develop counterarguments. Will commented: *“you know they’re there most of the time and… you might just totally ignore it… just like… you don’t wanna see the obvious… like the elephant in the room”* (24/NZE). Nick’s cognitive disengagement enabled new, and less worrying, explanations: *“I do sometimes think ‘that could be me one day’ but it doesn’t stop me… You think about it, then you put it outta your mind…. You think, well, there are non-smokers that get lung cancer as well and you rationalize it”* (NZE)*. “Rationalizing”* evolved into counterarguments where participants did not simply avoid PWLs, but actively challenged them.

### Defensiveness and Counterargument

The EPPM proposes that fear control may also include developing counter-arguments to manage feelings about PWLs rather than addressing the threat presented. Thus, despite comments that PWLs’ impact had waned, many participants reacted strongly to these and framed warnings as fake, insincere, and lacking credibility. They created alternative explanations, questioned PWL content, and privileged their own experiences above the so-called science shown. Freya explained: *“it’s a bit unrealistic… some of the pictures look so fake … it’s not actually real as such*” (33/NZE). Bridie argued PWLs were *“not realistic”* and lacked the authenticity that only lived experiences of smoking provided: *“there’s no information on it, there’s no nothing. They couldn’t* [have] *used better pictures or something different? I just don’t know who thought of that… did they even smoke?”* (26/NZE).

Others undermined PWLs’ trustworthiness by claiming they “scare-mongered” or were irrelevant. Amber argued: *“I know so many people who have smoked well into old age and been fine… it’s almost setting people up to be scared of something that might not happen”* (62/NZE). Several cited themselves as proof PWLs could not be correct. Penny stated: *“a lot of people say I’m quite short because my mother drunk and smoked with me as a baby, and I didn’t get fully developed … but I’ve never personally seen an example of smoking causing death or anything like that so it’s quite hard for it to hit home for me”* (25/NZE). Despite suggestions she experienced harms from her mother’s smoking, Penny amplified the threshold required from *“stuff happening”* to *“smoking causing death.”* The absence of evidence meeting this higher standard allowed her to reject links between her own lived experience and smoking’s harms.

While several participants rejected the messages PWLs communicated, others felt ambivalent about these. Jemma believed smoking had caused her grandfather’s death, but nonetheless felt “*50/50… not sure*” about PWLs because: *“I’ve known so many people in my life that have smoked, and still smoke, and I don’t think any of that has happened to them… but my grandad, he did die of fibrosis and emphysema from smoking his whole life, so yeah, I’m 50/50, I’m not sure”* (31/NZE). Despite her own experience, Jemma focused on people who appeared unaffected by smoking. Airini shared similar doubts; although she agreed that PWLs present credible risks, she queried their generalizability: “*smoking causes lung cancer and it probably does harm some babies, but I don’t think it does it to everyone*” (48/M). These participants created a disconnection between abstract and specific believability that allowed them to recognize risks while rejecting their personal relevance. Others exploited more fundamental disconnections. David stated: “*as a man, I’m not gonna have a baby so* [pregnancy PWL]*… doesn’t really affect me personally*” (34/NZE) and Harriet also preferred PWLs that did not apply to her: “*I just go for the ones that you know, harm children, ‘cause I don’t have children so it’s kind of irrelevant*” (57/NZE).

Some relocated responsibility to victims of the illness shown. Freya attributed serious gum disease to *“…more than just smoking… that’s from bad hygiene and probably other stuff as well”* (33/NZE,) while Harriet suggested a PWL featuring a diseased foot caused by circulatory problems was instead due to “*fungal problems… they’ve let their foot go like that … because they’re not looking after themselves”* (57/NZE). These arguments returned agency to people who smoke by implying they could avoid risks by *“looking after themselves.”*

Counterarguments also included attributing the risks shown to other etiologies. Hahana questioned the association between a PWL featuring a cancer patient (an Australian label she recalled) and smoking: *“the one lying on the bed, apparently, is not the person that died of lung cancer, or smoking, or whatever. It looked like an AIDS victim”* (35/M). Recalling the same image, Will commented: *“*[the] *guy with cancer in the hospital bed … that can really… happen to maybe anyone… even no matter how much they smoke… I think it’s just like your chance of cancer cells mutating”* (24/NZE). Framing harms as uncertain and likely to occur at random reduced fear and thus perceived pressure to quit.

However, while several participants used counterarguments to reassert their agency, others found agency elusive in a world replete with harm; their fatalistic perspective viewed smoking as just one of the many risks they encountered. Penny explained: *“everything’s gonna kill you one day… and unless you have an experience or another life to look out for, then I don’t think it really has much effect”* (25/NZE). Matthew’s apocalyptic narrative minimized smoking’s harms: *“I live in 2023. Everyone’s dying of health problems, you know? We had Covid, and we’ve got wars… So that’s* [PWL] *the least of my problems”* (39/NZE). Viewed alongside pandemics and warfare, smoking’s risks seemed minor and less threatening. Counterarguments took diverse forms and some evolved into reactance, where participants rejected perceived intrusions on their freedoms.

### Reactance and Reasserting Freedoms

Analyses of the EPPM also present reactance as a response used to control fear. We identified two forms of reactance: first, asserting smoking as less harmful than other practices and thus unfairly stigmatized; second, presenting PWLs as risible. Bridie illustrated the first approach: *“I just think it’s a bit strange… if you’re putting that* [PWLs] *on cigarettes, why aren’t you putting fat people on food?”* (26/NZE). Hahana made a similar case: *“I’d probably rather see these on an alcohol bottle than a packet of smokes. I reckon alcohol is more dangerous than smokes”* (35/M). By presenting smoking as a victim of disproportionate regulation, participants could diminish its risks.

Others used humor to diminish PWLs’ impact. Harriet deliberately misinterpreted a warning message to re-assert her agency: “*‘Smoking harms others’… it doesn’t harm me, so it is a bit of a joke*” (57/NZE), and thus unable to present a real threat. Maia explained: *“All my friends are smokers and they…look at these pictures and … they laugh, ‘Which one do you have this time?’ And they laugh about it… if you’re laughing about something, it’s not being effective towards you quitting”*(29/M). While fewer participants exhibited reactance, those who did felt aggrieved and resisted the cessation cues PWLs presented.

### Creating Connections: People versus Dismembered Bodies

Although participants had developed strategies to diminish the impact existing PWLs had on them, they did not dismiss these altogether and suggested improvements, including more frequent warning rotation, greater diversity, and stronger evidence. In particular, they argued warnings should consider them as whole people, rather than a collection of diseased organs, and engender empathy rather than fear. Potential new themes they proposed included the toll on personal relationships, the harms imposed on loved ones, the burden addiction created, and the difficult financial trade-offs smoking created. Olive compared the two approaches: “[Current PWLs have] *more shock value and more, ‘Oh my God,’… it points out like the premature ageing, cataracts, and mouth cancer and stuff. But I reckon seeing the kid’s drawing with the cigarette butts… it would… be like, ‘Oh my God, you know, I’ve got children’”*(28/NZE) Presenting smoking as potentially destroying children’s dreams evoked the same “*oh my God”* feeling but elicited a personal connection that physical risk images lacked.

Bridie thought new themes should be *“more real”* and suggested using testimonials where “*real people* [spoke]*, not just actors, people that have been through something that affected them purely from smoking”* (25/NZE). She felt this approach would inspire quitting instead of prompting avoidance or defensiveness. Freya called for a more “*positive approach… in a way of success stories… someone that’s been a smoker for 20, 50 years, and they’ve given up”*(33/NZE) and Tui suggested “*affirmations”* would motivate people (28/NZE). Participants wanted PWLs that inspired rather than terrified and suggested moving away from presenting diseased internal organs, which they found them difficult to recognize and lacking salience. David commented: *“Internal body… I’m not gonna see it, so I don’t really care… it’s just sort of out of sight, out of mind”* (34/NZE).

In addition, participants recommended fostering resilience, given quit attempts often failed, and suggested featuring people who had succeeded after several attempts. Others’ lived experiences created greater authenticity and seemed more likely to help them reimagine themselves as smokefree.

## Discussion

Our participants used varied strategies discussed in the EPPM literature to manage the fear and dissonance PWLs aroused.^16^ While a minority thought PWLs had remained visually impactful and believable, most did not notice, actively avoided, rationalized, or reacted against PWLs. Even those who argued PWLs had become less salient disliked seeing them and used avoidance or counterargument to manage negative affect.

The EPPM framework presents fear management as a maladaptive response to threats, such as those presented in PWLs, and our findings reinforce earlier studies documenting avoidance as a fear management strategy.^25–28^ We extend previous work by nuancing counterarguments used to manage fear^32^; these typically rejected “the science” PWLs presented in favor of participants’ lived experiences, where smoking’s risks were uncertain. Asserting themselves, or those they knew, as evidence PWLs were exaggerated, inaccurate or untruthful, not only reduced fear but returned agency to participants. While not always framed as reactions against perceived threats to their freedoms, most participants rejected PWLs, and many recommended a fundamental reorientation in their design and content.

Tired of warnings that framed them as patients-in-waiting, participants called for PWLs that encouraged and inspired change, for example, by presenting people who had quit successfully. Their call reflects concerns Hastings and colleagues outlined more than 2 decades ago, when they questioned social marketers’ growing reliance on fear appeals to foster behavior change.^45^ Although health promoters often use testimonials, those presented in PWLs typically feature adverse outcomes from smoking. Few countries (with the exception of Canada) offer tools to address the threat presented,^46^ despite evidence that empowering messages could increase self-efficacy, complement PWLs, and motivate cessation.^47^

Using “real people” who model adaptive threat control strategies could reduce participants’ frequent recourse to their own experiences,^39^ a strategy they used to rebut and rationalize PWLs that did not align with their day-to-day experiences. Recognizing people who smoke as sentient beings rather than at-risk organs, and offering threat control strategies, could increase willingness and ability to engage with warning information.

In addition, varying PWLs more frequently could address wear out,^48,49^ which may contribute to fear fatigue and message wear out, and stimulate defensive responses predicted by the EPPM.^20^ Unlike studies conducted shortly after plain packaging introduced larger PWLs, which could only assess the immediate impact these had, we have been able to observe and probe wear out. While we also detected avoidance, we found more evidence of counter-argument, a defensive response that may reflect rumination following sustained exposure to PWLs. Maintaining warning novelty using varied content and regular rotation would recognize the diversity in people’s lived experiences, increase the likelihood they see a message that resonates with their daily lives, and potentially reduce avoidance,^17–20^ counter argument^32,39^ and reactance.^35,36^ PWLs that offer support (eg, via efficacy messages) could foster adaptive threat control responses and reduce the strong fear control reactions existing PWLs elicit.^30^

Like all studies, ours has limitations. We recruited a diverse sample and, while diversity is a strength of qualitative work, we cannot estimate how effective PWLs that adopted participants’ advice would be relative to status quo approaches. Nor were we able to examine whether fear management strategies were associated with greater PWL impact.

Our sample comprised RYO tobacco users, a specific subset of people who smoke, though many participants had formerly or concurrently used tailor-made cigarettes. While we aimed to recruit a sample that was balanced by gender and ethnicity, we recruited fewer men and Māori participants than planned; nonetheless, we had sufficient information power to consider differences across gender and ethnicity. Māori researchers coded and oversaw data interpretation for Māori participants, thus ensuring an appropriate and insightful cultural lens. Nonetheless, future quantitative studies could allow generalizability and detailed comparisons by gender, ethnicity, and type of tobacco used. Given smoking places a heavy burden on Māori, future work led by Māori and using kaupapa Māori approaches may identify fear management strategies and alternative themes we did not detect.

Despite these limitations, our findings provide rich insights into how people using RYO tobacco interpret PWLs and reveal ideas that might foster greater engagement with these. Given the rising interest in tobacco endgame strategies, on-pack communications that engage people who smoke and inspire cessation attempts could play a crucial role in reducing smoking prevalence.

## Supplementary material

Supplementary material is available at *Nicotine and Tobacco Research* online.

ntae112_suppl_Supplementary_File_1

ntae112_suppl_Supplementary_File_2

ntae112_suppl_Supplementary_File_3

ntae112_suppl_Supplementary_File_4

## Data Availability

Our ethics approval (19/122) limited data access to members of the research team and participants gave informed consent to participate on this basis. We thus cannot share the transcripts but have provided additional quotations in a supplementary file to support and explain our interpretation of the data.
