# Supplementary material for: Managing Fear Responses: A Qualitative Analysis of Pictorial Warning Labels Five Years Post-Plain Packaging
Source: Nicotine Tob Res. 2024 Jun 6;27(6):1059–65. doi: 10.1093/ntr/ntae112 (PMC12095806; doi:10.1093/ntr/ntae112)
Supplement: ntae112_suppl_Supplementary_File_1 [file ntae112_suppl_supplementary_file_1.docx]

RYO Packaging Recruitment

Start of Block: Default Question Block

Q1.1
**Kia ora**
   **Thank you very much for your interest in our study.**
 
**Before we get in touch to let you know if we're able to set up an interview with you, we'd like you to answer a few short questions.**
 
**We'd like to recruit diverse participants and your answers will help us create a sample of people with different behaviours and characteristics.**

 **Please note: All the answers you give will be confidential to the research team.**

 **If you have any questions, please contact me at janet.hoek@otago.ac.nz, or phone 03 479 7692 or 021 150 6934**

| Page Break |  |
| --- | --- |

| 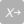 |
| --- |

Q1.2 **First, some questions about smoking
 Have you smoked at least 100 cigarettes in your life?**

- Yes (1)
- No (0)
- Don't know/Not sure (2)

Q1.3 **How often do you smoke now?**

- I don't smoke now (6)
- At least once a day (5)
- Not every day, but at least once a week (2)
- At least once a month (1)
- Less often than once a month (10)

End of Block: Default Question Block

Start of Block: Ineligibles

Q2.1
 
**Thank you for your interest in our study, unfortunately you are not eligible to participate.**

 **Please click NEXT to submit your questionnaire**

End of Block: Ineligibles

Start of Block: Current Smoking

Q3.1 **Which of these products do you smoke the most?**

- Tailor-made cigarettes (manufactured cigarettes from a packet) (1)
- Roll-your-owns made from loose tobacco (rollies) (2)
- Both tailor-mades and roll-your-owns (3)

Display This Question:

If How often do you smoke now? = At least once a day

And Which of these products do you smoke the most? = Tailor-made cigarettes (manufactured cigarettes from a packet)

Or How often do you smoke now? = At least once a day

And Which of these products do you smoke the most? = Both tailor-mades and roll-your-owns

Q3.2 **On average, how many tailor-made cigarettes do you smoke each day?**

________________________________________________________________

Display This Question:

If How often do you smoke now? = At least once a day

And Which of these products do you smoke the most? = Roll-your-owns made from loose tobacco (rollies)

Or How often do you smoke now? = At least once a day

And Which of these products do you smoke the most? = Both tailor-mades and roll-your-owns

Q3.3 **On average, how many roll-your-own cigarettes (rollies) do you smoke each day?**

________________________________________________________________

Display This Question:

If How often do you smoke now? = Not every day, but at least once a week

And Which of these products do you smoke the most? = Tailor-made cigarettes (manufactured cigarettes from a packet)

Or How often do you smoke now? = Not every day, but at least once a week

And Which of these products do you smoke the most? = Both tailor-mades and roll-your-owns

Q3.4 **On average, how many tailor-made cigarettes do you smoke each week?**

________________________________________________________________

Display This Question:

If How often do you smoke now? = Not every day, but at least once a week

And Which of these products do you smoke the most? = Roll-your-owns made from loose tobacco (rollies)

Or How often do you smoke now? = Not every day, but at least once a week

And Which of these products do you smoke the most? = Both tailor-mades and roll-your-owns

Q3.5 **On average, how many roll-your-own cigarettes (rollies) do you smoke each week?**

________________________________________________________________

End of Block: Current Smoking

Start of Block: Demographics

Q4.1 **Some questions about you
 How old are you?**

________________________________________________________________

Q4.2 **Which of the following best describes how you think of yourself?**

- Heterosexual or straight (1)
- Gay or lesbian (2)
- Bisexual (3)
- Other (4)
- I don't know (5)
- I prefer not to answer (6)

Q4.3 **Which ethnic group or groups do you belong to?
*Please tick all that apply***

- New Zealand European (1)
- Māori (2)
- Samoan (3)
- Cook Island Māori (4)
- Tongan (5)
- Niuean (6)
- Chinese (7)
- Indian (8)
- Other European (9)
- Other (such as Fijian, Korean) (10)
- Other (please specify) (11) __________________________________________________

| Page Break |  |
| --- | --- |

Q4.4 **What is your name?
 *Just a reminder that we will not share any information about you, or your answers with anyone outside our research team***

________________________________________________________________

Q18 **Are you living in Dunedin or Wellington?**

- Dunedin (1)
- Wellington (2)

Q4.5 **What is your mobile number?**

________________________________________________________________

Q4.6 **What is your email address?**

________________________________________________________________

Q4.7 **How would you prefer us to get in touch with you?**

- Mobile only (1)
- Email only (2)
- Either mobile or email is fine (3)

| Page Break |  |
| --- | --- |

Q4.8
**Thank you for your help with our research**  
**If you are eligible to take part in our study, one of the research team will be in touch with you soon**

 **Please click NEXT to submit your questionaire**

End of Block: Demographics
