# Supplementary material for: Managing Fear Responses: A Qualitative Analysis of Pictorial Warning Labels Five Years Post-Plain Packaging
Source: Nicotine Tob Res. 2024 Jun 6;27(6):1059–65. doi: 10.1093/ntr/ntae112 (PMC12095806; doi:10.1093/ntr/ntae112)
Supplement: ntae112_suppl_Supplementary_File_2 [file ntae112_suppl_supplementary_file_2.docx]

**Supplementary File 2: Participant Recruitment**

**Completed Interview**

**27 completed interview**

**33 invited to interview**

**Invited to Interview**

**Eligible and Willing to be Interviewed**

**49 willing to be interviewed**

**95 eligible contacts**

**151 total contacts**

**Recruited via social media**

**Initial Contacts**

**Eligible Contacts**

- 48 did not leave contact information
- 8 were ineligible (did not smoke RYO predominantly; could not be interviewed in person
- 32 could not be contacted (3+ attempts) after being sent an information sheet
- 4 were no longer interested
- 10 were initially responded but stopped responding (assumed no longer interested)
- 16 were from groups from which we had several participants (women, pākehā) and were held on file but not booked
- 5 did not attend booked interview; could not be reached for follow up
- 1 cancelled and could not be rebooked
