# Supplementary material for: Managing Fear Responses: A Qualitative Analysis of Pictorial Warning Labels Five Years Post-Plain Packaging
Source: Nicotine Tob Res. 2024 Jun 6;27(6):1059–65. doi: 10.1093/ntr/ntae112 (PMC12095806; doi:10.1093/ntr/ntae112)
Supplement: ntae112_suppl_Supplementary_File_3 [file ntae112_suppl_supplementary_file_3.docx]

**Supplementary File 3: Additional quotations illustrating themes**

| Avoidance | Physical avoidance | Harriet: “If you were that offended by it, you would just put your tobacco in another thing, so it’s pointless, I feel.”  Harriet: “At the end of the day people have cigarette cases that they drop their tobacco in.”  Hahana: “I gave- give it back to the lady at the shop, and she swaps it.”  Victor: “I’ll open her up and, yeah… not concentrate on it… I normally have a tin so pack’s only to chop it up… I use my tin cause that’s got all… everything I need in it.”  Maia: “But of course, there's all these marketing ploys out there so that you don't see that packaging. So you can get pouches that you can hide your tobacco in, containers that you can put your tailies in. So yeah…. I only use one because I got given it to me by my dad. So there is a little bit of sentimental value.” |
| --- | --- | --- |
|  | Familiarity and wearout | Cara: “I think we’ve kind of visually got used to them… they’re very graphic, we live in a very graphic world now, we’re exposed to a lot of images that have become part of our everyday lives so unfortunately I don’t think they encourage people to stop smoking anymore… people have got so used to images like this… we are constantly being exposed to thinks like this on television, on our social media… I think we’ve just got hardened to it.”  David: “I suppose it would vary from person to person but… most people who are smoking, they’re already been doing it for so long it’s just they’ve barely take notice of it, a lot of it… If it’s just something normal, you just ‘oh, like, whatever’ and just ignore it, sort of thing.”  Maia: “The same images have been in circulation kinda for years, so. Um, if you're gonna shock someone, I guess, you'd need to be changing it continuously with more and more horrific photos. And hope that maybe one gets to someone…. I would hope it work for someone. I know it wouldn't work for me.”  Maia: “To me, it's nothing shocking 'cause it's been out for so long. It's desensitisation. But of course, you know what it looks like because you interact with a tobacco pouch, you know, every single day.”  Victor: "This is all dead and buried, all that needs to go. It's just… Yeah, the- the colours, the warning, it is just... 'cause they're all the same. So, you don't see it. It's just, oh, yeah, just another packet of darts… when you see it a thousand times you just go, it doesn't have any meaning. If it's new and you go, ‘Ooh, that's different this time.’”  Victor: “I don’t really concentrate on it that much but I think the last sort of comment I had was in the smoking room, guys I work with they’re going ‘oh, look at that’ and they’re non-smokers… and I went ‘oh yeah’ but I won’t physically stand there and stare at the pictures because they are, yeah, yucky” [While Victor experiences wearout from current PWLs, when people who do not smoke comment on the labels, he is reminded of their impact.] |
| Avoidance (cont.) | Familiarity and wearout (cont.) | Fraser: “I do notice them, but I just sort of…not ignore them… but I already know what it does to my health and what it can do to other people and that, you know?”  Mathew: “I know for a fact my lungs are probably horrible and seeing pictures of it doesn’t make me wanna give up smoking cause I already knew it.”  Maia: “Zero effectiveness. Um, from my perspective, smokers know what the consequences are, we know that you can get lung cancer, brain cancer. It does all the damage to your skin, it can cause all these horrible things. Um, so putting it on a packaging, um, in my opinion, doesn't really give incentive to quit because you just get desensitised to it. Like, at the th-... When it first brought in, when I was younger , it was kind of, like, ‘Ew, this is kind of gross.’“ |
|  | Mental blocking | Nick: “When you buy a packet, like you get the packet and you…see it as you’re paying you might just think ‘oh my god’ then you, you just move on quickly because, well, what other choice have you got? To think about it all the time? Well, that’s not gonna help anyway, really, thinking about it all the time.”  Cara: “They’re graphic… I think people just block them out now.”  Victor: “I haven’t taken a lot of notice, I try not to because some of them are so graphic.”  Emere: “I mean, for me and like a couple of my mates, like the, it's, it's like, you know how you can selectively look at things. It's like you don't look at the packaging, it's already open and you're looking inside of it, you know, it's just like, oh, you know, already in there just going to get what I want. And then it's away again. So I don't think there's much that crosses people's minds. Personally, at least.”  Jemma: “I just try not to look at it… I just try to ignore it… I just want that rush from the smoke…it’s gross… but obviously it doesn’t put people off from smoking because they’re still buying them.” |
| Defensiveness and counterargument | Fake content | David: “just like sort of seeing a random picture with some person..”  Freya: “Like this one here, that looks like they’ve just got someone that smokes and just kind of used them as a model almost.”  Bridie: “They’re just sort of um, obviously random pictures that people have found and placed on packaging.”  Maia: “the lungs, like, from... I know from a medical perspective, I know they are lungs. But I've also had friends that have actually come to me and been like, ’Is that what your lungs actually look like?’ And I go, ’Yes.’ And they're like, ’That's not lungs." Just yeah, don’t really care (laughs).” |
|  | Not trustworthy, scaremongering | Matthew: “I don’t really pay attention to them because when they brought them out I was just like… we already know they are bad for our health. Already know their causes, effects; this is just like scaremongering as far as I’m concerned… it did work for some people, they gave up. But for people like me who smoke constantly… it does nothing.” |
| Defensiveness and counterargument (cont.) | Not trustworthy, scaremongering (cont.) | Rawiri: “To me, it was just a scare tactic for other people, as far as I'm concerned. Yeah. Yeah. So I never focused, yeah, on that. Um, I thought that, um, so long as I can afford it, I can do it. But, you know, looking at heart attacks, you know, causing heart attacks and stuff, I have seen them and I have, you know ... But, um, yeah, uh, I can visualise them and stuff, but really, they meant nothing to me.”  Bridie: “I don’t think they really feel anything. I don’t think they even take notice because it’s… a bit far-fetched to put on a pouch of tobacco.”  Maia: “I guess, though when it comes to that... the- the man dying on the tobacco pouch, I noticed that was in the news. I don't... can't remember how long ago. But I know from, like, my group of friends and that, m- it does reduce kind of how believable these are because there was that family saying, "That's our, yeah, son or brother or something. He didn't die of smoking. Why is his face on a packet?"… Yeah, not credible.” |
|  | Privileging own experience / Self as evidence to the contrary | Victor: “I don’t know if they’re smoking related… put it this way, I’ve never had feet like that (laughs).  Kylie: “That one [baby PWL] doesn’t affect me so much, I mean, I did smoke through all of my pregnancies and my children were all born good sized and so forth. But it is kind of disturbing seeing the little baby there but when you’ve had other things happen to your children, you’re sorta immune to it.  Hahana: [How believable?] “Well, that one, probably... I don't know if it's from smoking, though. …My cousin was a prem baby and he... His mother didn't smoke. So, that's not... Doesn't always happen. I smoked with my kid. Not much, but… He didn’t turn out little.”  Freya: “I know a lot of people that had babies that smoke and they’ve kind of turned out fine and … maybe asthma when they’re a bit older but that’s about it, yeah.” |
|  | Irrelevant | Harriet: “I just go for the ones that you know, harm children cause I don’t have children so it’s kind of irrelevant… as smokers, we don’t really care.”  Victor: “Which we all laugh at, because we go that’s not gonna affect us cause we’re not a pregnant woman.”  Amber: “And I think ‘well, I’m not pregnant, so that’s okay.’”  Maia: “From personal perspective, that one [Baby PWL] would not... does not bother me at all. … I don't like babies (laughs). So when I see that, it doesn't tug at any maternal heartstrings or anything.” |
|  | Disconnect between abstract and specific believability | Ngaio: [Q: How believable are PWLs] “Probably 50-50. Yeah. Probably 50-50 is where I’d go with it. Like, I believe it can happen, um, you just don’t believe it’ll happen to you. Or it, it’s gotta be a substantial amount of smoking before you’ll get there…. Just more my head saying that, yeah, kind of, you must be, have to be quite a hard, aggressive smoker for it to kind of have that effect. I, like, even people say to you, it can only take one. But, I think, because of my ego, it was, sort of, like, no, that’s, ’yeah, that’s not gonna happen to me,’ more than anything else.”  Freya: “I think smoking does make your lungs go like that… but then, I’ve never actually seen it in life or anything like that, so I wouldn’t really know to be honest.” |
| Defensiveness and counterargument (cont.) | Disconnect between abstract and specific believability (cont.) | Airini: “They're not nice. … I used to see lots of things similar to that anyway, in an ambulance, sort of things I've done in my past. So I can just... mm-hmm. Yeah. And I've also had cancer myself as well, so... And I still smoke. So, it's showing you something. Had chemo, had radiation, I'm still gonna smoke.”  Rawiri: “it worries me a bit about, um, breathing and that, because that's your breathing, and everything, you know? Sort of thing. I lost my father to lung cancer. He was 54 when he died. Wasn't enough to scare me though.” |
|  | Relocating responsibility | Maia: “I guess, the cosmetic one, that one 'cause no woman likes ageing (laughs). That one can be sometimes a little bit bothersome. But at the s- same time, from my perspective, that can all be managed with, um, high-end quality cosmetics, so. And I really don't know how smoking can kinda cause something that bad. [About foot PWL] Yeah. 'Cause I kinda feel like if you know something's going wrong with your foot, wouldn't you go to the doctor straight away (laughs)? And get it sorted.” |
|  | Other aetiologies | Amber: “It looks more like a diabetic effect really”  Freya: “it’s a bit unrealistic, I guess, cause some of the pictures look so fake, it’s like these ain’t images that are caused from that… there’s the teeth one as well… it’s like that’s more than just smoking that’s caused that issue… it’s like yeah nah, that’s not just from smoking … like the teeth one for instance, that’s from bad hygiene and probably other stuff as well.”  Bridie: “Where’s the research to show that that’s what it does? Because it’s like I said, that can happen from anything. Premature babies (laughs) can happen just on their own… and lung cancer can happen at any time… not really that believable… I mean, I guess some people have, you know, had these things happen to them..”  Awhi: “I've seen this here before. But other people, but the other people go, "Oh, I know someone who's got athlete's foot." They're talking about it, but they're not talking about it in the, in the fact that you can lose a toe, 'cause of lack of blood that's been blocked by your arteries from the smoking.”  Ngaio: “It's ugly, but, um, it's, like, sort of, I feel like there could've been other factors that could've kind of, added to it being that, that bad, yeah.”  Pete: “One of my mates, he’s getting older, he said he’s getting more circulation problems. And he’s like ’but it’s only mild' at the moment’… so being a, you know, kiwi bloke, it’s like… ‘oh I’m getting, so I’ll just do this’, you know so he’s just started doing things that’s helped a wee bit, get the circulation back up and the feet one, it’s just like ‘oh well, I’ll just do more twitches on my feet’ and cause we wear work boots, like I always have, we get that anyway so ‘ah, it’s just the bloody work boots’ so he’ll find a, he found a switch and I’ve done the same. It’s like ‘oh, bloody work boots, need to get out of them more often’ but it’s probably the smoking (laughs).”  Freya: “I feel that’s like more like gangrene or something like that, you know, like more different issues going on.”  Mathew: “But, you know, you just seen a baby, that could be caused by anything as far as I’m concerned.” |
| Defensiveness and counterargument (cont.) | Fatalism | Matthew: “That really does nothing. Like I don’t think they could really do anything else that would make me wanna give up smoking cause the people that wanted to give up smoking have already given up and the people that don’t really care about giving up smoking won’t be affected by any of this and they’re the ones like me that are still smoking now who need … a better incentive to give up smoking rather than you’re gonna die of health problems.”  Awhi: “I mean, it says, you know, ‘Damages your blood vessels.’ But it du- Yeah. 'Cause you're, you're, you're not really looking at what's been said, you're really looking at the pictures. You know, maybe if you did say with a lung one, right? For me, it would be down this pile, if I could see an incentive. This is what it is, this is what it's like after giving up. Is there's some hope? Because when I see that, I go, ’Oh well.’ Excuse my language. ’Oh well, I'm fucked. So what’s the point?’ So maybe if you did one before and then one that's been not smoking for so long, and it's improving. That might make me think, ’Oh shit.’  Rawiri: “I always thought, well, cancer, you don’t really see on the outside until it’s too late. So, you know, if you start showing ulcers or anything like that, you’re normally- the internal is already buggered and that.”  Matthew: “It doesn’t really make me think of anything at the present time cause it doesn’t change anything… it’s just pictures to me… I can see someone being mangled in a car crash but is that gonna stop me from driving? Probably not, so just makes me a bit more aware that it’s happening.” |
| Reactance and reasserting freedoms | Smoking unfairly singled out relative to other risks | Awhi: [talking about pregnancy with three kids] “But I still smoked though. And they turned out all right. I mean, the statistics are that they can, you know, be underweight, they can, you know, stunt their growth, blah, blah, blah. But it didn't really pay attention to the fact of, you're putting your baby at risk. At least I'm not drinking, so I, I kind of justified my addiction.” |
|  | PWLs a joke | Bridie: “It’s silly putting it on there…. I find that quite ridiculous (laughs) really.”  Kylie: “It is brought up in conversation around quite a few people around, you know, which ones they like and which ones they don’t like, and it’s quite funny.” |
|  |  | Harriet: “I thought that one’s good and someone I know changed it to ’Smoking is attractive’”  Cara: “People kind of, more crack a joke, um, with regards to some of them. You know, I know that I’ve had, um, male friends who, you know, the baby one… actually, no, in reference to probably to the pregnant one, you know ‘Luckily I’m a male and that won’t happen to me… not being male, not being pregnant, you know, so it’s not their problem.”  Victor: “Which we all laugh at, because we go that’s not gonna affect us cause we’re not a pregnant woman.” |
