# Supplementary material for: Managing Fear Responses: A Qualitative Analysis of Pictorial Warning Labels Five Years Post-Plain Packaging
Source: Nicotine Tob Res. 2024 Jun 6;27(6):1059–65. doi: 10.1093/ntr/ntae112 (PMC12095806; doi:10.1093/ntr/ntae112)
Supplement: ntae112_suppl_Supplementary_File_4 [file ntae112_suppl_supplementary_file_4.docx]

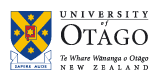


**INTERVIEW GUIDE**

**Roll-your-own Tobacco Packaging Study**

**Introduction**

Kia ora, my name is Janet; I arranged to meet with you to talk about the roll your own tobacco packaging study that we are undertaking.

**Interviewer to follow up earlier personal introduction as appropriate for participant. Offer to begin with karakia.**

I come originally from the North Island and grew up in Aorangi; I moved to Ōtepoti about ten years ago and love living near the moana. My father came to Aotearoa from the Netherlands as a teenager; he was looking for a new life after the war and was lucky to meet my mum, whose family came to Aotearoa from Ireland and England. My tane is a researcher like me and, between us, we have three daughters.

Is there anything you would like to tell me about yourself before the interview starts? **Allow participant to introduce her or himself and share any details they would like to make known.**

Would you like me to open our discussion with a karakia?

*Whakataka te hau ki te uru*

*Whakataka te hau ki te tonga*

*Kia mākinakina ki uta*

*Kia mātaratara ki tai*

*E hī ake ana te atakura*

*He tio, he huka, he hau hū*

*Tīhei mauri ora!*

Before we start I’d like to give you another copy of the information sheet we sent you earlier. Please take as long as you like to look through this form and let me know if you have any questions. While you’re looking at the information sheet, would you like me to make you a cup of coffee or tea?

**Ensure participant has time to read the information sheet.**

Do you have any questions you would like me to clarify? Please remember that you can ask me questions at any time during the session.

**Could I please turn on the audio recorder so that everything we discuss is captured? As you saw in the information sheet, I’m using an online transcribing service so our voices will be turned into words as the interview goes along.**

Now I have turned on the audio recorder, I will take you through the key points in the information sheet.

**Key points about consent to read out while the recorder is turned on:**

- As a participant in the research, you have the right to ask questions at any time; if there is something that is not clear, please tell me and I will do my best to answer your questions.
- You also have the right to decide if you would prefer not to answer some questions, so if we come to a topic you don’t feel comfortable discussing, please let me know and we will move on.
- You have the right to stop the interview and withdraw from the study at any time **up until the point the interview ends**. If you decide to withdraw from the study, there is no penalty.
- Thank you for giving permission to audio record this meeting and have it automatically transcribed word-for-word. Sometimes the software does not produce a good quality transcript and if that happens we will upload the audio file to Rev.com, which is based in the US and has people who transcribe from throughout the world. We would not know who would transcribe this interview but because some transcribers are based in New Zealand, there is a very small possibility that the person working on the transcript may recognise your voice but we think that is very unlikely.
- We would also like to take some photos of the RYO pouch designs you make as we go through the interview. We will not include you in any of these images, which will just be of the pouch elements you have selected and how you put these together. We will show you these images so you can check that you cannot be identified in these.
- We will use our best efforts to keep your responses confidential to members of the research team named on the Information Sheet.
- At the end of the interview I will ask you some short questions about yourself and ask if you would like a copy of your interview transcript, summary of your interview, and summary of the overall research findings.
- Just before we start, here is the gift voucher that we are offering everyone who takes part in our study. Could you please sign this form to show that you have received the voucher?

**Just a Final check, Do you have any questions before we get started?** **Please remember that you can ask me questions at any time during the session.**

**Could you please read and then sign the consent form. again, if you have any questions, please let me know.**

**If you need to take a break at any time, please let me know (acknowledge people may want to smoke)**

**Background information (10 minutes)**

I’d like to start by asking you some questions about your smoking:

1. **Can you tell me about when you first started smoking?**  **How did your smoking develop from there?** **What sort of tobacco did you smoke? Is that what you’ve always smoked?** Explore any switches between tailor made (TM) cigarettes and RYO tobacco.
2. **Can you tell me about your smoking now?**

- About how many cigarettes do you smoke each day (*or week, if non-daily*)?
- What do you think about RYO compared to TM? Probe perceived benefits of RYO tobacco, including: **harm, naturalness, addictiveness and cost-effectiveness (cheaper and easier to control consumption) etc.**

1. **What has smoking come to mean to you? What role does it play in your life?** Explore emotional engagement with smoking (stress relief and comfort versus control and loss of freedom).
2. **How do you think your smoking will develop in the future?** Explore future quitting intentions and possible reasons for wanting to quit. Explore factors that may impede quitting.

**Current PWLs (5 minutes)**

**Now, I’d now like to ask you about warnings used on New Zealand’s tobacco packages.**

1. Can you please tell me what warnings you’ve seen on New Zealand tobacco packaging? **Allow time for participants to think.**
2. What do you think makes you remember those warnings? **Explore role of visuals and what these mean to participants.**

**Now, I’m going to put some warning labels on the table [place images on table]; these are some warnings that are currently used in Aotearoa NZ:** (the images will include a harm to child image; an internal body part (lungs); an external body part (feet), and the social warning “Smoking is not attractive”).

1. Are there any that really catch your eye? (What is eye catching?) How believable are they? (What is not believable?) How relevant do you find them? (What is not relevant?)
2. What do you think people who smoke feel when they see these images? (Explore maladaptive /reactant responses reported)
3. Which images do you think would be most likely to encourage someone who smokes to think about quitting? What do you think makes that/these one(s) effective compared to the others?
4. How do you think these warnings could be improved so they are more effective at encouraging someone who smokes to think about quitting?
5. Are there other pictures or messages that you think would be more effective than these ones used currently? Can you tell me about these? What would they be like?

**Construct-a-pouch task (15-20 minutes)**

Thank you for letting me know what you think about those warning labels. Now, I’d like to ask you to create your own warning design using this pouch mock up [**give the pouch mock up to the participant**] and some images and messages I’ll show you. As I mentioned earlier, you might find some of the images upsetting. If that’s the case and you would like to move on or stop the interview, please let me know. Now, I’d like to ask you to look at these images (present set of warning images).

First, I’d like you to look at these images and put them into **three different piles** according to whether you think they are “*very likely to encourage someone who smokes RYO to think about quitting*”, “*possibly likely*”, or “*unlikely to encourage someone who smokes RYO to think about quitting*”.

**Place the printed label on the A3 sheet as each category is read out.**

Do you have any questions about what I’m asking you to do? (possible reassurance: “Things will become clear as we start working through the pictures.”) There are no right or wrong answers and everyone will respond differently.

1. **Here is the first image, which pile do you want to put that in?** Probe as relevant (e.g., if participant is hesitating “what’s going through your mind now” or if they move an image between piles “what led you to shift the image from **here to here?”**

*With your permission, I’d like to take a photo of the piles you’ve created and the images in each of these. I won’t include you in the photo and I’ll show you the photo after I’ve taken it so you can review it. Is that OK with you?*

Obtain verbal permission from participant before taking every photo; if participant moves images between categories, interviewer may need to take photos during the sorting process (e.g., say something like: “I’ll just capture that change you’ve made”).

Check how many images are in the “very likely” pile; if there are **fewer than five**, keep the “possible” pile on the table but place all the “unlikely” pile back into the envelope.

1. **Now, looking at the images you think are very likely (or very and possibly likely) to encourage someone smoking rollies to think about quitting, which one do you think would be most effective?**

Probe selection: What led you to pick this image? What is the main idea you think this image is communicating? Would you change anything to make it more effective?

Explore if participant seems to be considering two or more possible images “what was it about these images that made you think more about them? And what led you to choose the one you have selected?

**Please put the image onto this pouch that I have here (give participant blu tack to use).**

**Now, I’d like you to look at some messages that could go alongside the image you’ve chosen. Select set of messages that matches the key idea participant has associated with the image.**

If participant’s comments could correspond to different message sets; probe these with participant. You’ve described the image as communicating this idea [**REPEAT IDEA**], I’d like to give you some messages that correspond to your idea and would like to check I give you the best set. I have two sets that could match; one of these is about X and the other is about Y, which do you think best matches your idea?

**Choose the relevant set of messages according to the idea the participant has outlined.**

1. **I’d like you to sort these messages according to how likely you think they would be to encourage someone who uses RYO tobacco to think about quitting: very likely, possibly likely, or unlikely.**

Give text messages to participants in random order and probe as above to explore uncertainty and movement of text messages. Provide A4 sheet.

*Now, with your permission, I’d like to take a photo of the piles you’ve created and the messages in each of these. I won’t include you in the photo and it to you after I’ve taken it so you can review it. Is it OK if I take a photo now please?*

1. **Now, looking at the messages that you think are very likely to encourage someone using RYO tobacco think about quitting, which of these do you think would be most effective?** Probe selection: What led you to pick this message? How does it make you feel? How believable do you think it is? How relevant do you think it is to people who smoke?

Explore if participant seems to be considering two or more possible messages “what was it about these messages that made you think more about them? And what led you to choose the one you have selected? What is the main idea you think this message is sending? How does it fit with the image you have chosen?

**Please place the message on the pouch next to the image you chose and put there. Could I take a photo of your pouch, please?**

1. Thank you for selecting an image and message for your pouch. Could explain the story these tell?

- Thinking about people you know who smoke, how **believable** do you think this story would be to them? And how **relevant** do you think this story would be to **people who smoke? For Māori participants**, explore how relevant story is to **Māori** values and hopes.
- How does your pouch **differ from** RYO pouches available today? Probe: How important are those differences?
- How does looking at your pouch make you feel? Explore feelings such as fear, disgust, anger, sadness, disappointment, regret.
- **How would you feel if you bought RYO tobacco and were given this pouch?** Probe requesting another pouch, reading warning, avoiding warning, talking about warning with someone, thinking about quitting?

**Efficacy flap construction task (15-20 minutes)**

Now, I’d like to ask you to make some different designs; these will go on the inside of the pouch flap. Some countries use labels to help people thinking of trying to quit feel more confident that they can quit or more sure that quitting will benefit them or people they care about. What do you think of that idea? **Explore reaction, positive, negative, likely impact.** How likely do you think people would be to see information on this flap? And how likely do you think they would be to read it?

1. Here are some different explanations that could be included in these flaps. Could you take a moment to read through these and then sort them into three piles according to how helpful they would be to someone who smokes rollies and is thinking about quitting. The piles are “Very helpful”; “Helpful”, “Not helpful”. Here’s a sheet where you can place the messages **pass over A3 sheet.**

Allow time for participant to sort explanatory messages. Probe as relevant (e.g., if participant is hesitating “what’s going through your mind now” or if they move a message between piles “what led you to shift the message from **here to here”?**

*Now, with your permission, I’d like to take a photo of the piles you’ve created and the messages in each of these. I won’t include you and I’ll show you the photo after I’ve taken it so you can review it.*

1. **Now, looking at the explanations you think would be very helpful to someone using RYO tobacco and thinking about quitting, which of these do you think would be most helpful?** Probe selection: What was it that led you to pick this message? Explore if participant seems to be considering two or more possible images “what was it about these messages that made you think more about them? And what led you to choose the one you have selected? What is the main idea you think this message is sending? May need to probe to select relevant messages. **Please put the message on the inside flap of your pouch.**
2. **Now, I’d like you to look at some images that could go alongside the message you’ve chosen. Please sort these images according to how helpful** they would be to someone who smokes rollies and is thinking about quitting**. Please sort them into three piles:** “Very helpful”; “Helpful”, “Not helpful”. Here’s a sheet where you can place the images **pass over A3 sheet.** Probe as above to explore uncertainty and movement of images.

*Now, with your permission, I’d like to take a photo of the piles you’ve created; I won’t include you in the photo and it to you so you can review it.*

1. **Now, looking at the “very helpful” images, which of these do you think would be most helpful to someone smoking rollies and thinking about quitting?** Probe selection: What led you to pick this image? Explore if participant seems to be considering two or more possible images “what was it about these images that made you think more about them? And what led you to choose the one you have selected? What is the main idea you think this image is sending? How does it fit with the message you have chosen? Please place the image on the pouch by the message you placed there.
2. **Finally, I’d like you to look at some headlines that could link the image and message you’ve placed on your pouch. Please sort these headlines according to how well they connect the image and message you’ve chosen to help people smoking rollies and thinking about quitting.**

Give headlines to participants in random order and probe as above to explore uncertainty and movement of text messages. Provide A4 sheet with “very helpful”, “possibly helpful” “unhelpful” headings.

*Now, with your permission, I’d like to take a photo of the piles you’ve created and the messages in each of these. I won’t include you in the photo and it to you after I’ve taken it so you can review it. Is it OK if I take a photo now please?*

1. **Now, looking at the headlines that you think are very likely to help someone who smokes rollies and is thinking about quitting, which of these do you think would be most effective?** Probe selection: What led you to pick this headline? How does it connect the message and image you chose? How relevant do you think it is to people who smoke? How believable do you think it is?

Explore if participant seems to be considering two or more possible headlines “what was it about these messages that made you think more about them? And what led you to choose the one you have selected? What is the main idea you think this headline is sending? How does it fit with the message and image you have chosen?

1. Looking at the flap that you have created, could you explain the story you think it tells?

- Thinking about people you know who smoke, how **believable** do you think this story would be to them? And how **relevant** do you think this story would be to **people who smoke? For Māori participants**, explore how relevant story is to **Māori** values and hopes.
- How does your pouch **differ from** RYO pouches available today? Probe: How important are those differences?
- How does looking at your pouch make you feel? Explore feelings such as pride, aspiration, aroha, empowerment.
- **How would you feel if you bought RYO tobacco and were given this pouch?** Probe reading flap, avoiding flap, talking about flap with someone, thinking about quitting?

1. Overall, how do you think the pouch you have created compares with your current pouch of tobacco? What are the main differences? How does the story from the pouch you created compare to the story in the pouch you bought? How important are those differences?
2. Thinking about the two pouches, how much do you think they each motivate people to quit? How empowered do you think people will feel after seeing **current pouch** compared to **novel** pouch?
3. Finally, is there anything you can think of that would improve the images you’ve chosen or the messages that you’ve selected?

**Conclusion**

These are all the questions I wanted to ask you. Do you have any other comments you’d like to make?

Finally, I just have a short questionnaire for you to complete. Like the rest of our discussion, the information you provide will be completely confidential and only members of the research team will be able to access it.

**PROVIDE RESPONDENT WITH BACKGROUND QUESTIONNAIRE AND COLLECT AND CHECK ON COMPLETION**

**Check that participant has signed the gift voucher receipt (should have been completed initially)**

End meeting with karakia if meeting started with a karakia

Unuhia, unuhia

Unuhia ki te uru tapu nui

Kia wātea, kia māmā, te ngākau, te tinana, te wairua i te ara takatā

Koia rā e Rongo, whakairia ake ki runga

Kia tina! TINA! Hui e! TĀIKI E!
